# Editorial for the Special Issue “Infectious Diseases: Emerging Diagnostic Methods, Updated Treatment Protocols and New Antimicrobial Agents”

**DOI:** 10.3390/biology14091245

**Published:** 2025-09-11

**Authors:** Andreas G. Tsantes, Rozeta Sokou, Vanessa Bellou, Dimitrios V. Papadopoulos

**Affiliations:** 1 Microbiology Department, “Saint Savvas” Oncology Hospital, 11522 Athens, Greece; 2 Laboratory of Haematology, Blood Bank Unit, “Attiko” Hospital, School of Medicine, National and Kapodistrian University of Athens, 12462 Athens, Greece; 3 Department of Neonatology Aretaieio Hospital, National and Kapodistrian University of Athens, 11528 Athens, Greece; sokourozeta@gmail.com; 4 Department of Emergency Medicine, General Hospital of Arta, 47100 Arta, Greece; mdnessa@gmail.com; 5 2nd Department of Orthopaedic Surgery, “Konstantopouleio” General Hospital, National and Kapodistrian University of Athens, 14233 Athens, Greece; di_papadopoulos@yahoo.gr

Infectious diseases are one of the greatest challenges for global health, with significant socioeconomic implications. Although recent decades have witnessed remarkable scientific advances, the pace of microbial evolution and the rise in antimicrobial resistance have placed unprecedented pressure on healthcare systems worldwide [1,2]. Therefore, research should be focused on the development of innovative diagnostic modalities, novel therapeutic approaches, and new antimicrobial agents [3,4]. The Special Issue “Infectious Diseases: Emerging Diagnostic Methods, Updated Treatment Protocols and New Antimicrobial Agents” includes a set of six high-quality studies that highlight the new challenges in infectious disease management.

The work by Abdel-Hadi et al. is focused on the potential of nanotechnology as a novel field of research against multidrug-resistant pathogens. This study is one of the most innovative articles in this Special Issue [5]. Their research on the myco-synthesis of silver nanoparticles with Fusarium oxysporum demonstrates the potent antibacterial and antifungal properties of these particles. This study highlights that such green nanotechnologies could be useful in developing novel antimicrobial agents. Specifically, the authors showed that these nanoparticles cause structural damage to fungal hyphae and lead to considerable zones of inhibition against multidrug-resistant bacteria. The clinical implications of this study are significant, particularly as multidrug resistance continues to rise globally, and such bio-inspired nanoparticles may eventually find their place as adjuncts or even replacements in the antimicrobial armamentarium.

The association between infection, immunity, and coagulation is evaluated in the study by Papadogeorgou et al. The authors of this study assessed the hemostatic profiles of neonates who suffered from sepsis and provided evidence that levels of ADAMTS-13 are lower in these neonates [6]. Reporting for the first time a deficiency of ADAMTS-13 in septic neonates, the authors suggested that this could open avenues for novel therapeutic approaches, including ADAMTS-13 replacement as an adjunctive therapy, to improve the survival rate of neonates with this devastating condition. The clinical implications of this study are highly significant, reflecting the translational strength of this study. Moreover, the authors of this study underscored that infection should be managed as a part of a whole spectrum of derangements; thus, a more holistic approach could result in improved outcomes.

Accurate and prompt pathogen identification is of great significance for the successful management of complex infections, especially in fragile populations. Kapoor et al. compared two diagnostic modalities, polymerase chain reaction (PCR) and urine culture in cases of complex urinary tract infections [7]. Based on the results of this study, which included a large population of more than 3000 patients, PCR may be a more accurate method in detecting polymicrobial and fastidious organisms compared to traditional urine cultures. However, the authors highlighted that molecular methods such as PCR should be not used alone, but in conjunction with traditional culture methods, since both these methods can be more accurate in identifying different subsets of pathogens. This is essential since, while molecular diagnostics have been a great evolution in microbiology by providing rapid and sensitive results, the importance of culture-based methods for antimicrobial susceptibility testing and epidemiological surveillance cannot be overstated. The study offers a practical framework for clinicians, advocating combined approaches that maximize diagnostic yield and improve therapeutic decision-making in difficult-to-treat urinary tract infections.

The genomic aspect of antimicrobial resistance is investigated in the article by Borgio et al., which evaluates a highly virulent and multidrug-resistant Enterococcus faecalis strain isolated from a long-term hospitalized patient, using whole-genome sequencing to characterize the pathogen [8]. The alarming findings of this study are focused on specific mutations that can provide this pathogen with resistance mechanisms to powerful antimicrobial agents such as linezolid. Moreover, this study highlights the high virulence of E. faecalis, since, based on the molecular evaluation of this strain, E. faecalis pathogens have specific mechanisms that allow them to adhere, form biofilms, and evade immune responses. By reporting these genomic features, the study provides crucial data for surveillance and reinforces the urgent need for novel antibiotics and targeted infection control strategies. It also serves as a stark reminder that resistance is not merely a microbiological issue, but a clinical reality with profound implications for patient outcomes.

Acanthamoeba keratitis is a devastating local infection of the cornea, more common in people wearing lenses, that poses both diagnostic and treatment challenges, since it is associated with potential irreversible vision loss. The review by Shareef et al. focused on recent advances in the diagnosis and management of Acanthamoeba keratitis [9]. Historically, this infection was associated with poor outcomes due to delays in diagnosis and limited treatment options. However, recent developments, including sensitive molecular techniques, antibody-based assays, and machine-learning–assisted diagnostics, allow for a more accurate and timely diagnosis of this entity. Moreover, the introduction of oral miltefosine and the use of single-drug topical regimens are promising advances in treatment options, offering more effective and accessible solutions. By consolidating evidence from about 90 recent publications, this review provides a comprehensive update that is of great value to clinicians and researchers, while also illustrating how technological innovation, whether molecular diagnostics or artificial intelligence, can reshape clinical paradigms.

The article by Tsantes et al. addresses one of the most complex challenges in orthopedic oncology: periprosthetic infections of oncological prostheses [10]. These complex infections are particularly devastating due to the already vulnerable patient population and the high stakes of limb-salvage procedures. The review consolidates current knowledge on epidemiology, risk factors, microbiology, and treatment strategies, while also emphasizing recent advances in molecular diagnostics such as PCR and next-generation sequencing. The authors thoroughly explain how biofilm development functions as a primary disease-causing process that makes these infections extremely challenging to eliminate. The authors also emphasize the importance of surgical treatment through one-stage or two-stage revisions in cases of prosthetic joint infections, while discussing biofilm-resistant prosthetic materials as a preventive measure. This review unites microbiological analysis with surgical expertise and technological knowledge to create a valuable resource for medical professionals who treat prosthetic joint infections.

The studies included in this collection cover a broad spectrum of research areas, ranging from nanotechnology-based antimicrobial agents and molecular diagnostics to the pathophysiology of sepsis, rare ocular infections, and the intricate problem of periprosthetic joint infections. Together, these articles provide fundamental insights and practical perspectives that will advance both infectious disease research and clinical care. The Guest Editors express their appreciation to the authors for their important work and to the reviewers for their expert evaluations which maintained the high scientific standards of this Special Issue. The findings presented in this work should motivate additional research and stimulate professional discussions between disciplines to achieve improved patient results worldwide.

## References

[1] Sokou R., Palioura A.E., Kopanou Taliaka P., Konstantinidi A., Tsantes A.G., Piovani D., Tsante K.A., Gounari E.A., Iliodromiti Z., Boutsikou T. (2024). Candida auris Infection, a Rapidly Emerging Threat in the Neonatal Intensive Care Units: A Systematic Review. J. Clin. Med..

[2] Gartzonika K., Politi L., Mavroidi A., Tsantes A.G., Spanakis N., Priavali E., Vrioni G., Tsakris A. (2023). High prevalence of clonally related ST182 NDM-1-producing Enterobacter cloacae complex clinical isolates in Greece. Int. J. Antimicrob. Agents.

[3] Laxminarayan R., Sridhar D., Blaser M., Wang M., Woolhouse M. (2016). Achieving global targets for antimicrobial resistance. Science.

[4] Toumasis P., Tsantes A.G., Tsiogka A., Samonis G., Vrioni G. (2024). From Clinical Suspicion to Diagnosis: A Review of Diagnostic Approaches and Challenges in Fungal Keratitis. J. Clin. Med..

[5] Abdel-Hadi A., Iqbal D., Alharbi R., Jahan S., Darwish O., Alshehri B., Banawas S., Palanisamy M., Ismail A., Aldosari S. (2023). Myco-Synthesis of Silver Nanoparticles and Their Bioactive Role against Pathogenic Microbes. Biology.

[6] Papadogeorgou P., Boutsikou T., Boutsikou M., Pergantou E., Mantzou A., Papassotiriou I., Iliodromiti Z., Sokou R., Bouza E., Politou M. (2023). A Global Assessment of Coagulation Profile and a Novel Insight into Adamts-13 Implication in Neonatal Sepsis. Biology.

[7] Kapoor D.A., Holton M.R., Hafron J., Aljundi R., Zwaans B., Hollander M. (2024). Comparison of Polymerase Chain Reaction and Urine Culture in the Evaluation of Patients with Complex Urinary Tract Infections. Biology.

[8] Borgio J.F., AlJindan R., Alghourab L.H., Alquwaie R., Aldahhan R., Alhur N.F., AlEraky D.M., Mahmoud N., Almandil N.B., AbdulAzeez S. (2023). Genomic Landscape of Multidrug Resistance and Virulence in Enterococcus faecalis IRMC827A from a Long-Term Patient. Biology.

[9] Shareef O., Shareef S., Saeed H.N. (2023). New Frontiers in Acanthamoeba Keratitis Diagnosis and Management. Biology.

[10] Tsantes A.G., Altsitzioglou P., Papadopoulos D.V., Lorenzo D., Romano C.L., Benzakour T., Tsukamoto S., Errani C., Angelini A., Mavrogenis A.F. (2023). Infections of Tumor Prostheses: An Updated Review on Risk Factors, Microbiology, Diagnosis, and Treatment Strategies. Biology.

